# Supplementary material for: Systems Biology Analysis Reveals Eight SLC22 Transporter Subgroups, Including OATs, OCTs, and OCTNs
Source: Int J Mol Sci. 2020 Mar 5;21(5):1791. doi: 10.3390/ijms21051791 (PMC7084758; doi:10.3390/ijms21051791)
Supplement: Supplementary file 1 [file ijms-21-01791-s001.zip › ijms-730724-SupplementaryFiguresS1-6 Tables S2-7.pdf]

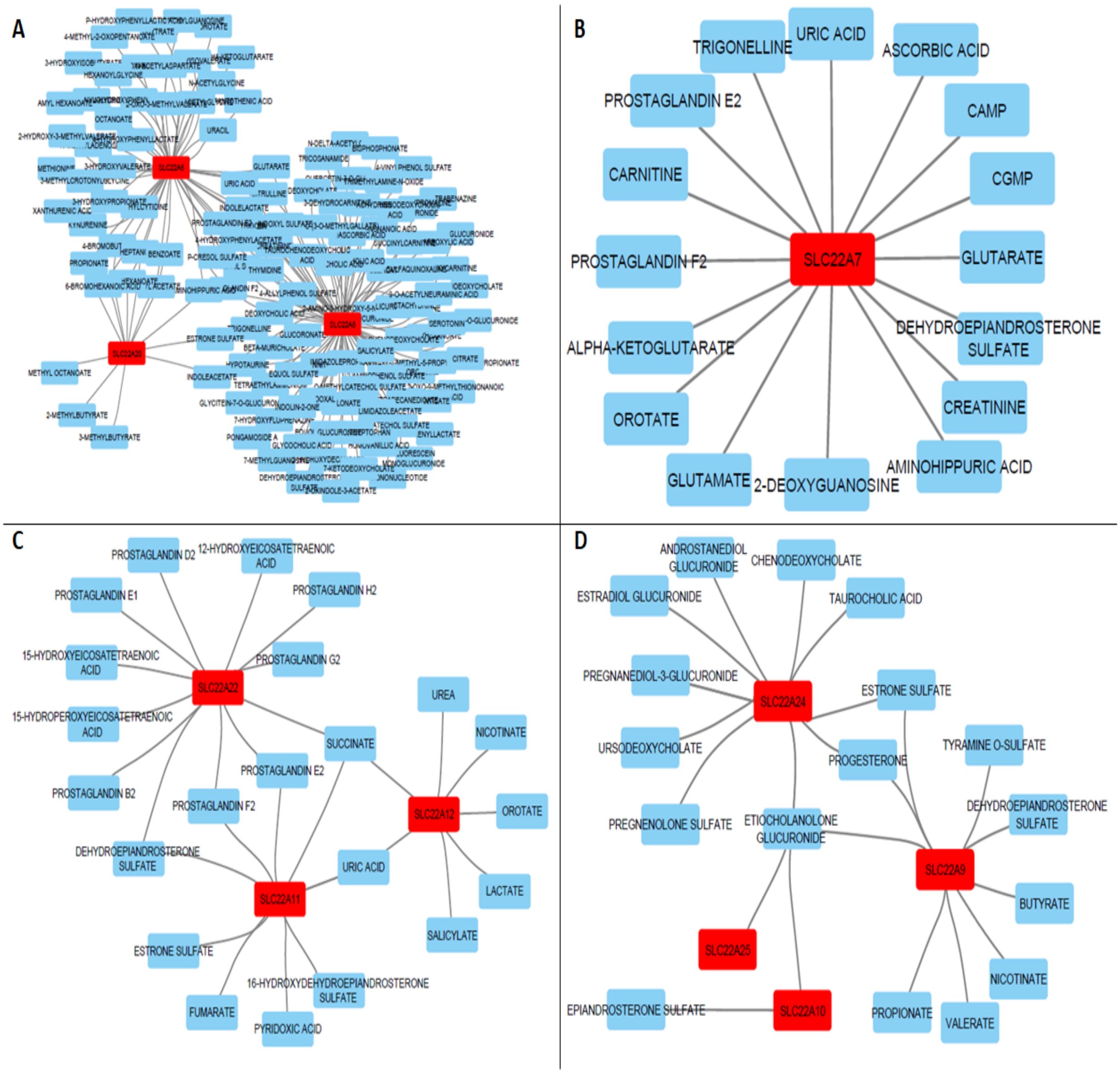

Figure S1: OATS1-4 Subgroups. The previously defined OAT major subclade was reconstructed into smaller groups based on additional support. A) OATS1 (SLC22A6, SLC22A8, SLC22A20). B) OATS2 (SLC22A7). C) OATS3 (SLC22A11, SLC22A12, Slc22a22). D) OATS4 (SLC22A9, SLC22A10, SLC22A24, SLC22A25). In each panel, the SLC22 family members are red nodes, the metabolites are blue nodes, and the edges connecting them are evidence of interaction between the protein and the metabolite.

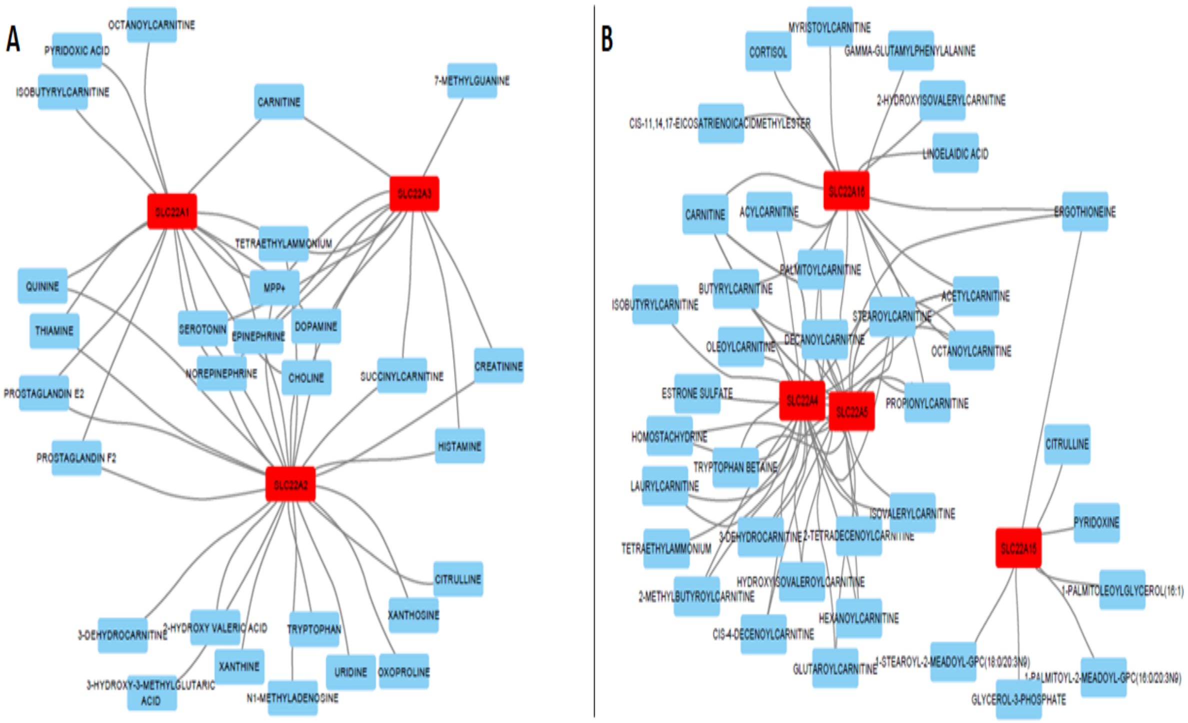

Figure S2: OCT and OCTN/OCTN-related subgroups. The previously defined OCT major subclade was reconstructed into new groups based on additional support. A) OCT (SLC22A1, SLC22A2, SLC22A3). B) OCTN/OCTN-Related (SLC22A4, SLC22A5, SLC22A15, SLC22A16). In each panel, the SLC22 family members are red nodes, the metabolites are blue nodes, and edges connecting them are evidence of interaction between the protein and the metabolite.

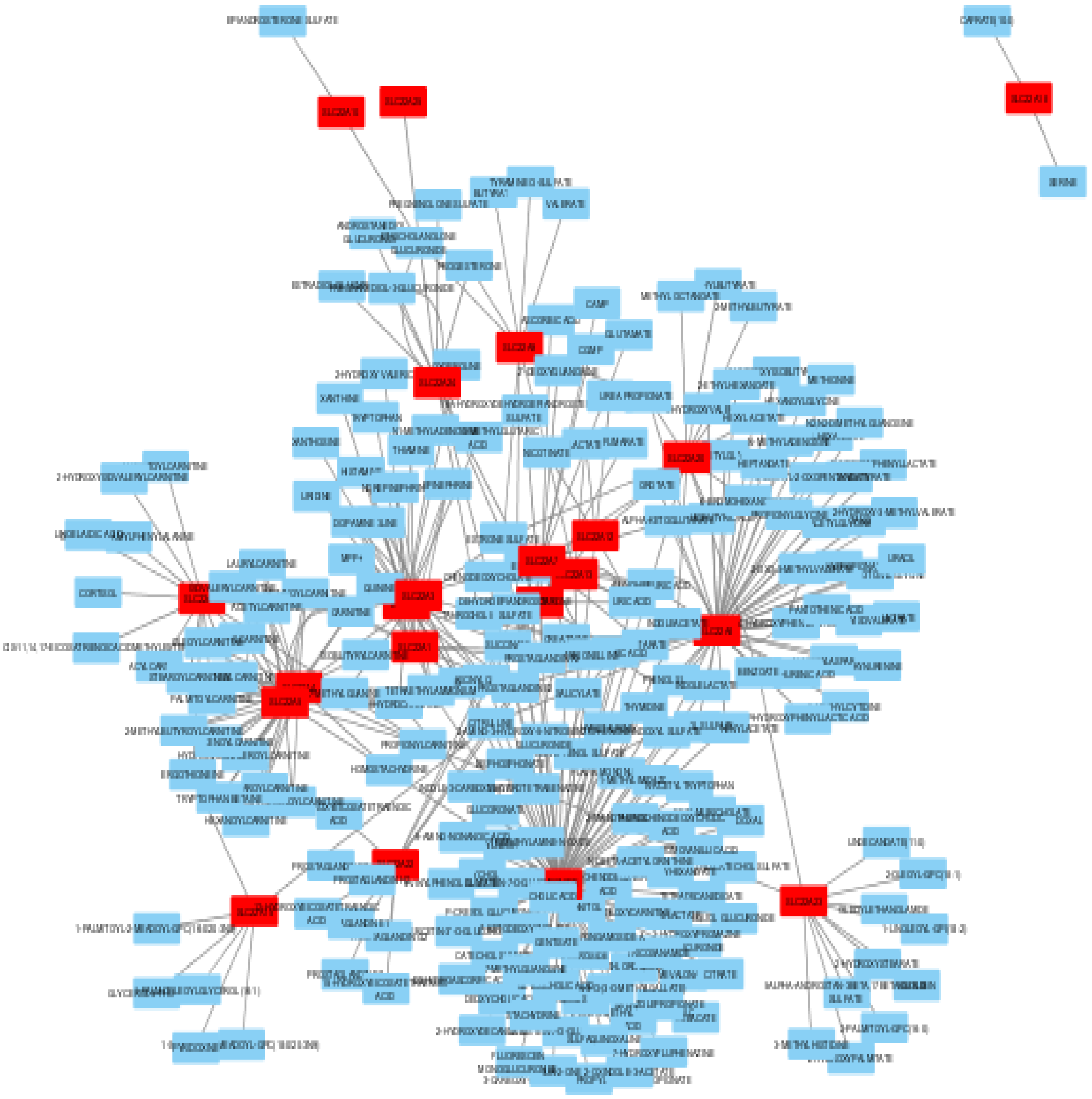

Figure S3: Total SLC22 Network. Every found association is represented by an edge connecting a metabolite to a transporter. SLC22 transporters are red nodes, and metabolites are light blue nodes.

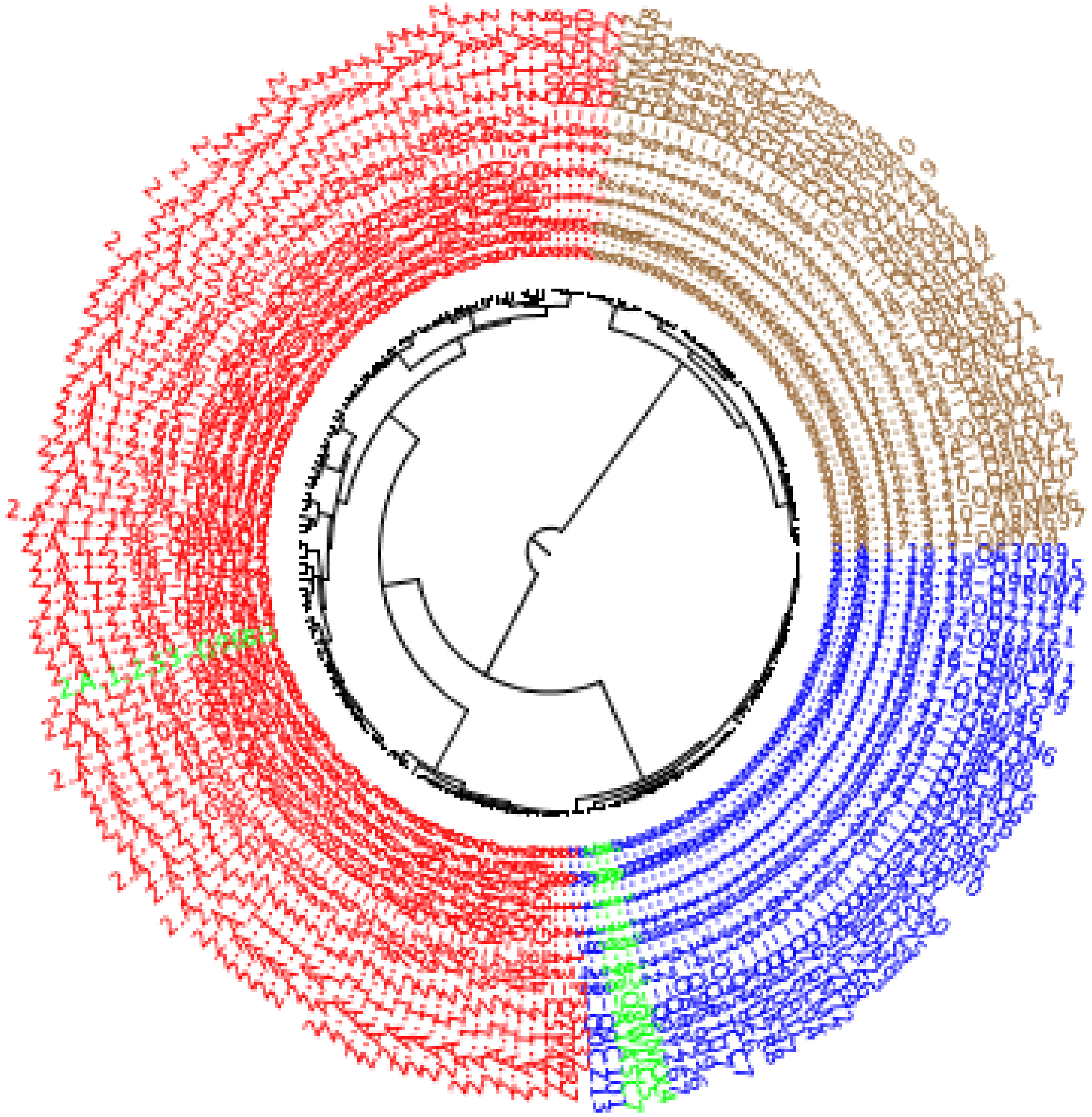

Figure S4: SSearch36 homology results show Slc22a18 within the DHA family. The DHA family is shown in red, with SLC22A18 highlighted in green. The SLC22 family is shown in blue, with the other Oat-related subclade members (SLC22A17, SLC22A23, and SLC22A31) highlighted in green. The POT transporter family outgroup is shown in brown.

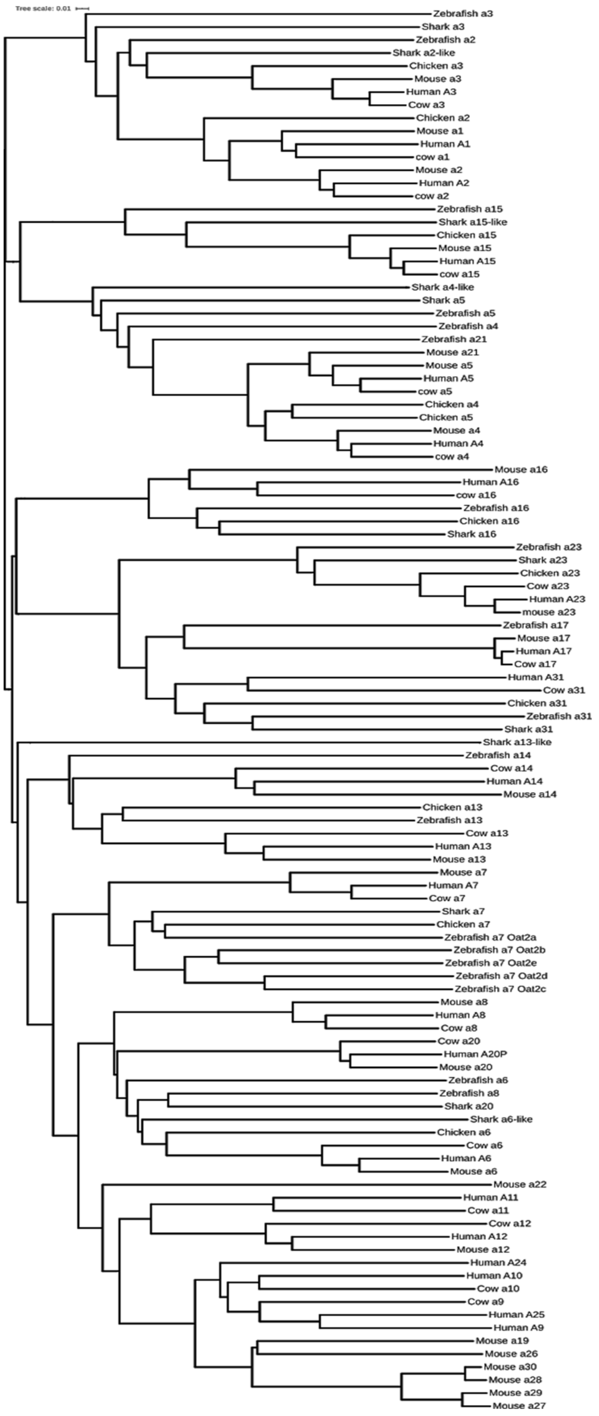

Figure S5: MAFFT multiple sequence alignment. Phylogenetic tree of previously defined OAT subclade. 90 sequences were aligned using MAFFT and viewed on the Interactive Tree of Life (iTOL). Branch lengths indicate the number of amino acid changes per residue between the sequences and the most recent ancestor. This analysis supports the formation of 4 functionally distinct subgroups from the OAT subclade.

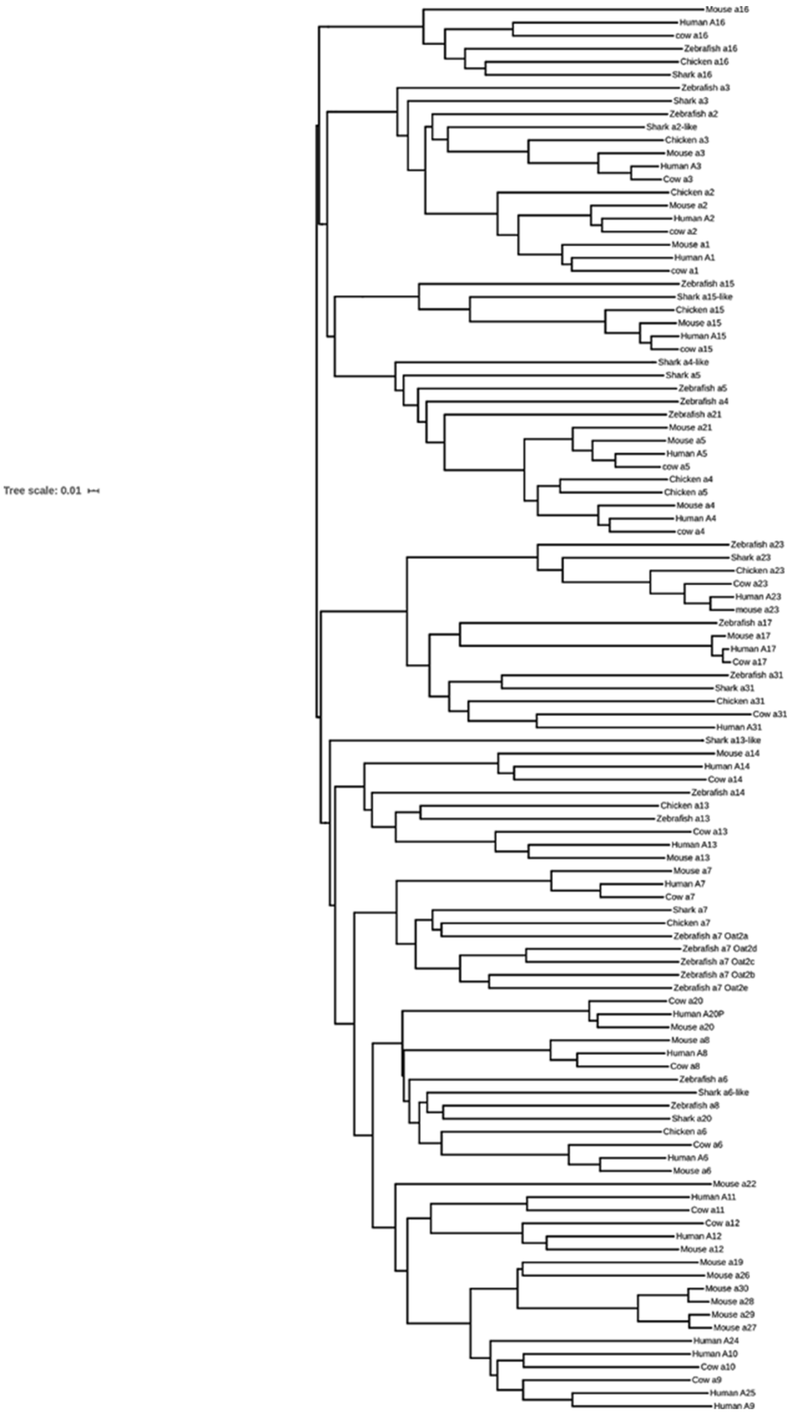

Figure S6: Clustal-Omega multiple sequence alignment. All SLC22 sequences from human, mouse, cow, chicken, shark, and zebrafish were aligned using Clustal-Omega. Tree was visualized using iToL.

	Motif	Region	% Present	MEME#	p-value	Sequence
Oat subclade	A	1-20	0.97	12	2.27E-20	MAFNDLLQQVGGVGRFQQIQ
	B	33-52	0.99	2	1.23E-22	SHNTLQNFTAAIPTHHCRPP
	C	67-86	0.99	5	8.88E-19	LPRDRQGQPESCLRFTSPQW
	D	99-118	0.99	1	7.82E-21	TGATEPCTDGWIYDNSTFPS
	E	123-142	0.99	3	3.01E-20	EWDLVCSHRALRQLAQSLYM
	F	143-162	1	6	5.34E-18	VGVLLGAMVFGYLADRLGRR
	G	177-196	1	13	1.09E-20	TCAAFAPNFPIYCAFRLLSG
	H	230-249	0.99	10	5.99E-21	YSLGQFLLAGVAYAVPHWRH
	I	261-275	0.95	11	2.59E-16	FFIYSWFFIESARWH
	J	279-298	0.95	7	6.4E-17	GRLDLTLRALQRVARINGKR
	K	328-338	0.86	15	1.69E-09	ELLRCPTLRHL
	L	349-368	0.97	9	1.28E-21	TSFAYYGLVMDLQGFGVSIY
	M	376-395	0.97	16	5.25E-18	AVDLPAKLVGFLVINSLGRR
	N	423-437	0.86	14	2.1E-16	RTSLAVLGKGCLAAS
	O	438-457	0.93	8	2.02E-23	FNCIFLYTGELYPTMIRQTG
	P	503-522	0.97	4	8.46E-20	LLPETLGQPLPDTVQDLESR
	Motif	Region	% Present	MEME #	p-value	Sequence
Oat subgroup 1	1	163-176	1	12	2.92E-22	KVLILNYLQTAVSG
	2	211-229	1	9	4.90E-25	VEWMPIHTRACVGTLIGYV
	3	250-252	1	6	1.39E-23	LQL
	4	255-260	1	5	1.14E-23	SAPFFA
	5	299-307	1	7	1.65E-23	EEGAKLSME
	6	346-348	1	3	2.32E-24	WFA
	7	369-375	1	10	4.42E-22	LIQVIFGAVDLPAKLVG
	8	415-422	1	13	1.34E-21	IPQDQSIV

Table S2: Evolutionarily conserved Oat subclade and Oat subgroup 1 motifs. Table describing the Oat Subclade-specific (blue) and OATS1-specific (Slc22a6, Slc22a8, and Slc22a20; red) conserved motifs that have been identified within the hOAT1 (hSLC22A6) sequence. This table lists the motif letter, prevalence, MEME#, p-value and amino acid sequence of hOAT1 associated with the particular motif letter.

	Motif	Region	% Present	MEME #	p-value	Sequence
Oat Subclade	A	43485	0.97	12	5.3E-18	MGFEELLEQVGGFGPFQLRN
	B	33-52	0.99	2	4.6E-17	LHFLLPIFLAAVPAHRCALP
	C	69-88	0.99	5	7.5E-16	LPREPDGTLSSCLRFAYPQA
	D	107-126	0.99	1	5.3E-20	EPATVPCSQGWEYDHSEFSS
	E	133-152	0.99	3	1.1E-16	QWDLVCEQKGLNRAASTFFF
	F	153-172	1	6	8.6E-21	AGVLVGAVAFGYLSDRFGRR
	G	187-206	1	13	1.2E-11	LASAASVSYVMFAITRTLTG
	H	240-259	0.99	10	4.4E-17	WTGGVMLLALVGYLIRDWRW
	I	271-285	0.95	11	1.3E-14	GILSLWWVPESARWL
	J	289-318	0.95	7	1.5E-14	GHVKEAHRYLLHCARLNGRP
	K	336-346	0.86	15	1.4E-12	DLFRTPRLRHI
	L	357-376	0.97	9	1.1E-17	VNFSYYGLSLDVSGLGLNVY
	M	384-403	0.97	16	1.3E-15	AVELPSKLLVYLSVRYAGRR
	N	431-445	0.86	14	9.3E-12	STVLAVMGKAFSEAA
	O	446-465	0.93	8	6.6E-21	FTTAYLFTSELYPTVLRQTG
	P	511-530	0.97	4	8.5E-20	LLPETRQAQLPETIQDVERK
	Motif	Region	% Present	MEME #	p-value	Sequence
Oat subgroup 2	1	127-131	1	4	4.61E-26	TIATE
	2	207-211	1	14	1.05E-23	SALAG
	3	214-233	1	9	3.18E-25	IIVMPLELEWLDVEHRTVAG
	4	286-288	1	2	2.51E-26	LTQ
	5	347-356	1	5	2.29E-26	SLCCVVVWFG
	6	377-383	1	10	4.05E-23	QTQLLFGAVELPSK
	7	466-481	0.93	15	2.44E-22	MGLTALVGRLGGSLAP

Table S3: Evolutionarily conserved Oat subclade and Oat subgroup 2 motifs. Table describing the Oat Subclade-specific (blue) and OATS2-specific (Slc22a7; red) conserved motifs that have been identified within the hOAT2 (hSLC22A7) sequence. This table lists the motif letter, prevalence, MEME#, p-value and amino acid sequence of hOAT2 associated with the particular motif letter.

	Motif	Region	% Present	MEME #	p-value	Sequence
Oat Subclade	A	1-20	0.97	12	6.05E-20	MAFSELLDLVGGLGRFQVLQ
	B	33-52	0.99	2	7.53E-19	TQSMLENFSAAVPSHRCWAP
	C	77-96	0.99	5	7.02E-23	IPPGPNQRPHQCRRFRQPQW
	D	110-129	0.99	1	4.49E-25	EADTEPCVDGWVYDRSIFTS
	E	134-153	0.99	3	3.36E-19	KWNLVCDSHALKPMAQSIYL
	F	154-173	1	6	2.27E-18	AGILVGAAACGPASDRFGRR
	G	188-207	1	13	1.50E-17	TAAAFAPAFPVYCLFRFLLA
	H	241-260	0.99	10	1.64E-13	FSFGHGLTAAVAYGVRDWTL
	I	272-286	0.95	11	1.06E-17	CFLYSWWLAESARWL
	J	290-309	0.95	7	1.41E-16	GRLDWGLQELWRVAAINGKG
	K	339-349	0.86	15	1.34E-07	TLLRMPGLRFR
	L	360-379	0.97	9	2.17E-16	FGFTFFGLALDLQALGSNIF
	M	387-406	0.97	16	1.92E-17	VVDIPAKMGALLLLSHLGRR
	N	434-448	0.86	14	2.87E-11	RSALAVLGLGGVGAA
	O	449-468	0.93	8	4.70E-19	FTCITIYSSELFPTVLRMTA
	P	514-533	0.97	4	2.33E-20	LLPETQSLPLPDTIQDVQNQ
	**Motif**	**Region**	**% Present**	**MEME #**	**p-value**	**Sequence**
Oat subgroup 3	1	53-57	1	3	7.75E-25	LLDNS
	2	98-109	0.85	15	5.94E-19	LLDPNATATSWS
	3	130-132	1	1	4.79E-27	TIV
	4	174-182	1	8	3.61E-22	LVLTWSYLQ
	5	185-187	1	12	2.53E-21	VMG
	6	261-263	1	13	7.90E-20	LQL
	7	313-332	0.95	14	3.34E-20	DTLTPEVLLSAMREELSMGQ
	8	380-382	0.95	10	1.65E-21	LLQ
	9	417-433	0.9	16	2.86E-23	GLCILANTLVPHEMGAL

Table S4: Evolutionarily conserved Oat subclade and Oat subgroup 3 motifs. Table describing the Oat Subclade-specific (blue) and OATS3-specific (Slc22a11 and Slc22a12; red) conserved motifs that have been identified within the hURAT1 (hSLC22A12) sequence. This table lists the motif letter, prevalence, MEME#, p-value and amino acid sequence of hOAT3 associated with the particular motif letter.

	Motif	Region	% Present	MEME #	p-value	Sequence
Oat Subclade	A	1-20	0.97	12	2.41E-15	MDFDEILHHVGDSGRFQICM
	B	33-52	0.99	2	7.07E-18	PHDVLENFTAAIPAHHCSIN
	C	76-95	0.99	5	9.03E-21	IPMGPNQKPEQCRRFRYTQW
	D	109-128	0.99	1	1.34E-20	ELETEPCLDGWTYDHSVFTS
	E	133-152	0.99	3	1.64E-14	EWDLVCDFQSFKYYAQATSL
	F	153-172	1	6	1.13E-13	AGHLVSCPLSGIISDRFGRK
	G	187-206	1	13	3.93E-16	TYCAFAPNFSVYCVLRFLLS
	H	240-259	0.99	10	7.52E-15	NSIGQGVLGGLAYVISDWHL
	I	271-285	0.95	11	3.33E-12	FFVLFCWVPESVRWL
	J	289-308	0.95	7	1.12E-17	GKTDQAWKELQRIASINGKK
	K	338-348	0.86	15	1.17E-07	DIFINPLIRKI
	L	359-378	0.97	9	4.39E-12	ELFSFVGLLLDVQLLGKNMF
	M	386-405	0.97	16	1.20E-12	AIDVPSKSLTYFTIRNVSRR
	N	433-447	0.86	14	4.15E-10	RTIIFILGKGCFAAF
	O	513-532	0.97	4	7.45E-16	FLPETFNLPHTDIIKDMEKR
	**Motif**	**Region**	**% Present**	**MEME #**	**E-value**	**Sequence**
Oat subgroup 3	1	53-57	1	3	2.67E-16	LDNSR
	2	129-131	1	2	1.62E-24	TIV
	3	173-181	1	8	1.57E-16	PLLMYCSLA
	4	184-186	1	12	4.47E-18	AVG
	5	260-262	1	13	2.32E-17	LQL
	6	267-270	1	4	1.73E-18	PFFI
	7	312 -331	1	14	8.23E-14	QNLTTEDLRSKLKKDVNSTG
	8	379-381	1	11	3.17E-15	LTQ
	9	416-432	1	16	2.34E-12	GSCITITIFISEEMYVL
	10	450-469	1	10	5.19E-13	ISTTYINELSPVELRSTLNG

Table S5: Evolutionarily conserved Oat subclade and Oat subgroup 3 motifs. Table describing the Oat Subclade-specific (blue) and OATS3-specific (Slc22a11, Slc22a12, and Slc22a22; red) conserved motifs that have been identified within the mOat-pg (mSlc22a22) sequence. This table lists the motif letter, prevalence, MEME#, p-value and amino acid sequence of mOat-pg associated with the particular motif letter.

Group	Motif	Region	% Present	MEME #	p-value	Sequence
Oat Subclade	A	1-20	0.97	12	4.45E-17	MAFQDLLGHAGDLWRFQILQ
	B	33-52	0.99	2	1.24E-21	LHFMLENFTAFIPGHRCWVH
	C	77-96	0.99	5	1.62E-24	IPLDSNMRPEKCRRFVHPQW
	D	110-129	0.99	1	5.65E-21	DADMEPCVDGWVYDRISFSS
	E	134-153	0.99	3	5.02E-20	EWDLVCDSQSLTSVAKFVFM
	F	154-173	1	6	1.32E-19	AGMMVGGILGGHLSDRFGRR
	G	188-207	1	13	3.15E-19	TCAALAPTFLIYCSLRFLSG
	H	241-260	0.99	10	3.35E-14	SGIAFMTLAGLAFAIRDWHI
	I	272-286	0.95	11	1.16E-13	IFLTSSWLLESARWL
	J	290-309	0.95	7	9.21E-19	NKPEEGLKELRKAAHRSGMK
	K	361-380	0.97	9	9.18E-17	NFMAYFGLNLHVQHLGNNVF
	L	388-407	0.97	16	2.04E-13	AVILLANCVAPWALKYMNRR
	M	435-449	0.86	14	1.11E-09	REVLATLGLGASALA
	N	450-469	0.93	8	1.77E-13	NTLAFAHGNEVIPTIIRARA
	O	515-534	0.97	4	2.09E-19	LLPETRNKPLFDTIQDEKNE
	Motif	Region	% Present	MEME #	p-value	Sequence
Oat subgroup 4	1	53-57	1	3	6.06E-27	ILDND
	2	62-76	0.96	14	1.06E-16	NDTGALSQDALLRIS
	3	100-109	1	13	8.06E-17	HLNGTFPNTS
	4	130-133	1	1	7.76E-25	TIVT
	5	174-182	1	8	1.39E-23	FVLRWCYLQ
	6	261-268	1	10	9.11E-23	LQLVVSVP
	7	287-289	0.92	5	2.20E-25	IIN
	8	310-315	0.92	15	1.28E-16	NARDTL
	9	333-352	0.92	12	2.41E-18	KKKPSLCEMLHMPNICKRIS
	10	418-434	0.88	16	2.51E-21	AICLLAIIFVPQEMQTL
	11	488-507	0.92	11	1.70E-23	MILSVYSPPLPWIIYGVFPF

Table S6: Evolutionarily conserved Oat subclade and Oat subgroup 4 motifs. Table describing the Oat Subclade-specific (blue) and OATS4-specific (Slc22a9, Slc22a10, Slc22a24, Slc22a25, Slc22a19, Slc22a26, Slc22a27, Slc22a28, Slc22a29, and Slc22a30; red) conserved motifs that have been identified within the hOAT7 (hSLC22A9) sequence. This table lists the motif letter, prevalence, MEME#, p-value and amino acid sequence of hOAT7 associated with the particular motif letter.

	Motif	Region	% Present	MEME #	p-value	Sequence
Oat Subclade	A	1-20	0.97	12	6.05E-20	MSFQELLNQVGSLGRFQILQ
	B	33-52	0.99	2	1.44E-21	PHIAMENFTAAIPNHRCWVP
	C	77-96	0.99	5	1.59E-23	IPLDSNLRPEKCRRFAQPQW
	D	110-129	0.99	1	8.62E-25	EPDTEPCVDGWVYDRSNFLS
	E	134-153	0.99	3	3.89E-20	EWDLVCESQALNSVTKFSFM
	F	154-173	1	6	3.19E-18	IGLFIGGIICGHLSDRLGRK
	G	188-207	1	13	1.96E-20	TCVAFAPSFFIYCSLRFLAG
	H	241-260	0.99	10	5.73E-15	PNIGYMISAGLAFLFRIWHH
	I	272-286	0.95	11	1.06E-13	FLILTRWLSESARWL
	J	290-309	0.95	7	8.47E-24	NKPQKGLKELRKVAHMNGMK
	K	338-348	0.86	15	1.76E-11	DLFHTSILRKR
	L	359-378	0.97	9	1.08E-13	FTVSIFGLAVHLQHLSSNII
	M	386-405	0.97	16	2.05E-12	ALAILVSVIGPFVLNHIGRR
	N	448-467	0.93	8	1.10E-12	VGVSRLHTNELLPTTLRATA
	O	513-532	0.97	4	2.90E-19	LLPETKNQPLPDSTHDVGND
	Motif	Region	% Present	MEME #	p-value	Sequence
Oat subgroup 4	1	53-57	1	3	2.87E-27	ILDND
	2	58-76	1	8	1.88E-24	TASDNGSRILSQDDLLRIS
	3	130-133	1	6	7.81E-26	TIVT
	4	174-178	1	12	1.44E-23	FILTC
	5	181-187	1	7	2.59E-26	LQFAITE
	6	215-234	1	14	2.03E-24	VNSHLLMLEWTSPKFLTMM
	7	261-268	1	9	9.82E-25	LQLTMSVP
	8	287-289	1	5	2.26E-26	IVT
	9	310-316	1	10	1.41E-25	NSGDLTM
	10	331-337	1	11	2.08E-24	KTKPSLR
	11	406-412	1	15	2.00E-24	ITYLVLM
	12	489-508	1	13	2.12E-24	ATYYANMPWIFYGGFSIFNA

Table S7: Evolutionarily conserved Oat subclade and Oat subgroup 4 motifs. Table describing the Oat Subclade-specific (blue) and OATS4-specific (Slc22a19, Slc22a26, Slc22a27, Slc22a28, Slc22a29, and Slc22a30; red) conserved motifs that have been identified within the mouse Slc22a27 sequence. This table lists the motif letter, prevalence, MEME#, p-value and amino acid sequence of mSlc22a27 associated with the particular motif letter.
